# Genome-wide analysis implicates microRNAs and their target genes in the development of bipolar disorder

**DOI:** 10.1038/tp.2015.159

**Published:** 2015-11-10

**Authors:** A J Forstner, A Hofmann, A Maaser, S Sumer, S Khudayberdiev, T W Mühleisen, M Leber, T G Schulze, J Strohmaier, F Degenhardt, J Treutlein, M Mattheisen, J Schumacher, R Breuer, S Meier, S Herms, P Hoffmann, A Lacour, S H Witt, A Reif, B Müller-Myhsok, S Lucae, W Maier, M Schwarz, H Vedder, J Kammerer-Ciernioch, A Pfennig, M Bauer, M Hautzinger, S Moebus, L Priebe, S Sivalingam, A Verhaert, H Schulz, P M Czerski, J Hauser, J Lissowska, N Szeszenia-Dabrowska, P Brennan, J D McKay, A Wright, P B Mitchell, J M Fullerton, P R Schofield, G W Montgomery, S E Medland, S D Gordon, N G Martin, V Krasnov, A Chuchalin, G Babadjanova, G Pantelejeva, L I Abramova, A S Tiganov, A Polonikov, E Khusnutdinova, M Alda, C Cruceanu, G A Rouleau, G Turecki, C Laprise, F Rivas, F Mayoral, M Kogevinas, M Grigoroiu-Serbanescu, P Propping, T Becker, M Rietschel, S Cichon, G Schratt, M M Nöthen

**Affiliations:** 1 Institute of Human Genetics, University of Bonn, Bonn, Germany; 2 Department of Genomics, Life and Brain Center, University of Bonn, Bonn, Germany; 3 Institute of Physiological Chemistry, Philipps-University Marburg, Marburg, Germany; 4 Institute of Neuroscience and Medicine, Research Center Juelich, Juelich, Germany; 5 Institute for Medical Biometry, Informatics and Epidemiology, University of Bonn, Bonn, Germany; 6 Institute of Psychiatric Phenomics and Genomics, Ludwig-Maximilians-University Munich, Munich, Germany; 7 Department of Genetic Epidemiology in Psychiatry, Central Institute of Mental Health, Medical Faculty Mannheim/University of Heidelberg, Heidelberg, Germany; 8 Department of Biomedicine, Aarhus University, Aarhus, Denmark; 9 Institute for Genomics Mathematics, University of Bonn, Bonn, Germany; 10 National Center Register-Based Research, Aarhus University, Aarhus, Denmark; 11 Division of Medical Genetics, Department of Biomedicine, University of Basel, Basel, Switzerland; 12 German Center for Neurodegenerative Diseases, Bonn, Germany; 13 Department of Psychiatry, Psychosomatic Medicine and Psychotherapy, University Hospital Frankfurt am Main, Frankfurt, Germany; 14 Max Planck Institute of Psychiatry, Munich, Germany; 15 Munich Cluster for Systems Neurology (SyNergy), Munich, Germany; 16 University of Liverpool, Institute of Translational Medicine, Liverpool, UK; 17 Department of Psychiatry, University of Bonn, Bonn, Germany; 18 Psychiatric Center Nordbaden, Wiesloch, Germany; 19 Center of Psychiatry Weinsberg, Weinsberg, Germany; 20 Department of Psychiatry and Psychotherapy, University Hospital Carl Gustav Carus, TU Dresden, Dresden, Germany; 21 Department of Psychology, Clinical Psychology and Psychotherapy, Eberhard Karls University Tübingen, Tübingen, Germany; 22 Institute of Medical Informatics, Biometry and Epidemiology, University Duisburg-Essen, Essen, Germany; 23 Cologne Center for Genomics, University of Cologne, Cologne, Germany; 24 Department of Psychiatry, Laboratory of Psychiatric Genetics, Poznan University of Medical Sciences, Poznan, Poland; 25 Department of Cancer Epidemiology and Prevention, Maria Sklodowska-Curie Memorial Cancer Centre and Institute of Oncology Warsaw, Warsaw, Poland; 26 Department of Epidemiology, Nofer Institute of Occupational Medicine, Lodz, Poland; 27 Genetic Epidemiology Group, International Agency for Research on Cancer, Lyon, France; 28 Genetic Cancer Susceptibility Group, International Agency for Research on Cancer, Lyon, France; 29 School of Psychiatry, University of New South Wales, Randwick, NSW, Australia; 30 Black Dog Institute, Prince of Wales Hospital, Randwick, NSW, Australia; 31 Neuroscience Research Australia, Sydney, NSW, Australia; 32 School of Medical Sciences, Faculty of Medicine, University of New South Wales, Sydney, NSW, Australia; 33 Queensland Institute of Medical Research, Brisbane, QLD, Australia; 34 Moscow Research Institute of Psychiatry, Moscow, Russian Federation; 35 Institute of Pulmonology, Russian State Medical University, Moscow, Russian Federation; 36 Russian Academy of Medical Sciences, Mental Health Research Center, Moscow, Russian Federation; 37 Department of Biology, Medical Genetics and Ecology, Kursk State Medical University, Kursk, Russian Federation; 38 Institute of Biochemistry and Genetics, Ufa Scientific Center of Russian Academy of Sciences, Ufa, Russian Federation; 39 Department of Genetics and Fundamental Medicine, Bashkir State University, Ufa, Russian Federation; 40 Department of Psychiatry, Dalhousie University, Halifax, NS, Canada; 41 National Institute of Mental Health, Klecany, Czech Republic; 42 Montreal Neurological Institute, McGill University, Montreal, QC, Canada; 43 Department of Human Genetics, McGill University, Montreal, QC, Canada; 44 McGill Group for Suicide Studies and Douglas Research Institute, Montreal, QC, Canada; 45 Department of Psychiatry, McGill University, Montreal, QC, Canada; 46 Département des sciences fondamentales, Université du Québec à Chicoutimi (UQAC), Chicoutimi, QC, Canada; 47 Department of Psychiatry, Hospital Regional Universitario, Biomedical Institute of Malaga, Malaga, Spain; 48 Center for Research in Environmental Epidemiology, Barcelona, Spain; 49 Biometric Psychiatric Genetics Research Unit, Alexandru Obregia Clinical Psychiatric Hospital, Bucharest, Romania

## Abstract

Bipolar disorder (BD) is a severe and highly heritable neuropsychiatric disorder with a lifetime prevalence of 1%. Molecular genetic studies have identified the first BD susceptibility genes. However, the disease pathways remain largely unknown. Accumulating evidence suggests that microRNAs, a class of small noncoding RNAs, contribute to basic mechanisms underlying brain development and plasticity, suggesting their possible involvement in the pathogenesis of several psychiatric disorders, including BD. In the present study, gene-based analyses were performed for all known autosomal microRNAs using the largest genome-wide association data set of BD to date (9747 patients and 14 278 controls). Associated and brain-expressed microRNAs were then investigated in target gene and pathway analyses. Functional analyses of *miR-499* and *miR-708* were performed in rat hippocampal neurons. Ninety-eight of the six hundred nine investigated microRNAs showed nominally significant *P*-values, suggesting that BD-associated microRNAs might be enriched within known microRNA loci. After correction for multiple testing, nine microRNAs showed a significant association with BD. The most promising were *miR-499*, *miR-708* and *miR-1908*. Target gene and pathway analyses revealed 18 significant canonical pathways, including brain development and neuron projection. For *miR-499*, four Bonferroni-corrected significant target genes were identified, including the genome-wide risk gene for psychiatric disorder *CACNB2*. First results of functional analyses in rat hippocampal neurons neither revealed nor excluded a major contribution of *miR-499* or *miR-708* to dendritic spine morphogenesis. The present results suggest that research is warranted to elucidate the precise involvement of microRNAs and their downstream pathways in BD.

## Introduction

Bipolar disorder (BD) is a severe neuropsychiatric disorder with an estimated lifetime prevalence of 1%.^1^ BD is characterized by recurrent episodes of mania and depression, and shows a heritability of ~70%.^2^ Molecular genetic candidate studies and—more recently—genome-wide association studies (GWAS) have identified the first BD susceptibility genes.^3, 4, 5, 6, 7^ However, the disease pathways and underlying regulatory networks remain largely unknown.^8^

Accumulating evidence suggests that microRNAs (miRNAs) are implicated in the biological pathways that regulate brain development and synaptic plasticity.^9, 10^ This in turn suggests their possible involvement in the pathogenesis of several psychiatric disorders,^11, 12^ including BD.^13, 14^ Studies of the post-mortem brain tissue of BD patients have demonstrated altered miRNA expression profiles in the prefrontal cortex.^13, 14^

The miRNAs are a class of 21–25-nucleotide small noncoding RNAs. In the nucleus they are transcribed by RNA polymerase II to primary miRNA (pri-miRNA) transcripts, which are double-stranded stem loop structures comprising 100–1000 nucleotides.^15, 16^ Approximately 50% of all vertebrate miRNAs are processed from the introns of protein-coding genes or from genes encoding other noncoding RNA classes. However, miRNAs can also be encoded in intergenic regions.^17^

The pri-miRNAs are then processed by the Drosha-DGCR8 complex to precursor miRNAs.^18, 19^ These precursor miRNAs are 60–70 nucleotides in length. The precursor miRNAs are exported to the cytoplasm, where they are cleaved into ∼20-base pair (bp) mature miRNAs by the Dicer enzyme.^16, 20^ The mature miRNAs are incorporated into the RNA-induced silencing complex, which then targets distinct sets of messenger RNAs (mRNAs).^21^

The miRNAs control the expression of their target genes by binding to target sites within the mRNAs, typically in their 3′ untranslated regions.^22, 23^ A region of 2–7 or 2–8 consecutive nucleotides from the 5′ end of the mature miRNA forms the seed region, which is crucial for the recognition of the target genes.^24^ In general, each miRNA controls up to several hundred target mRNAs, whereas one mRNA target can be subjected to synergistic regulation by multiple miRNAs.^25, 26^ In consequence, miRNAs integrate different intracellular signals and regulate a number of signaling pathways.^27, 28^ Interestingly, the miRNA regulatory effect itself has been shown to be a heritable trait in humans.^29^

The hypothesis that miRNAs are implicated in BD is also supported by the results of the largest GWAS of BD to date.^6^ In this study, a single-nucleotide polymorphism (SNP) in an intergenic region flanking *MIR2113* on chromosome 6q16.1 was the eighth strongest finding. However, no significant enrichment of BD-associated genes within the known or predicted targets of *MIR2113* was observed.^6^

Several studies have investigated the role of single miRNAs in the development of psychiatric disorder,^30, 31, 32^ including BD.^33^ However, to our knowledge, no systematic, genome-wide analysis of miRNA-coding genes has yet been performed. The aim of the present study was, thus, to determine whether common variants at any of the known miRNA loci contribute to the development of BD.

## Materials and methods

### Sample description

The gene-based tests were performed using data from our previous GWAS of BD (9747 patients and 14 278 controls).^6^ This GWAS data set combined data from Canada, Australia and four European countries (MooDS) with the GWAS results of the multinational Psychiatric Genomics Consortium (PGC).^3^ The study was approved by the respective local Ethics Committees. Written informed consent was obtained from all participants.^6^

### Genome-wide miRNA association analysis

For the gene-based analyses, a set-based testing approach adapted from the versatile gene-based test for GWAS^34^ was used. This algorithm is obtainable upon request. The chromosomal positions of all miRNAs (*n*=718) were obtained from miRBase release 13.0.^35^ This release contains a high confidence set of miRNAs for which detailed information about miRNA function and predicted target genes is available. Using the summary statistics, gene-wide *P*-values were calculated for all 636 autosomal miRNAs and their ±20 kilobase (kb) flanking sequences. Twenty-seven of these miRNA loci contained no common SNP. Therefore, gene-wide *P*-values were obtained for 609 miRNAs.

The applied statistical algorithm is described in more detail in the article by Liu *et al.*^34^ Briefly, SNPs within these boundaries were grouped together, and a set-based test statistic was calculated as the sum of the *χ*^2^ one degree of freedom association *P*-values within the miRNA. The test statistic was compared with simulated test statistics from the multivariate normal distribution. An empirical miRNA-based *P*-value was calculated as the proportion of simulated test statistics above the observed test statistic. For the purposes of the present study, the 10% most significant SNPs for each miRNA were summarized. The calculated gene-based *P*-values were Bonferroni-corrected for multiple testing according to the number of investigated miRNAs (*n*=609).

As different reference panels were used for the imputation of the MooDS and PGC genotype data (1000 Genomes Project, February 2012 release, and HapMap phase 2 CEU, respectively), we used simulated test statistics on the basis of an intermarker linkage disequilibrium (LD) structure as derived from the HapMap phase 2 population genotypes. However, for miRNAs that showed a significant association with BD after Bonferroni correction, we also calculated gene-based tests based on 1000 Genomes Project phase 3 population genotypes.

Inflation of the observed and expected *P*-values for different SNP subcategories (SNPs in miRNA loci, SNPs in genes and intergenic SNPs) was defined as the degree of deviation from the expected uniform distribution in the quantile–quantile (Q–Q) plot and tested for significance using Fisher's exact test (one-sided) for different *P*-value thresholds. Only LD-pruned SNPs (*r*^2^<0.8) were used for the enrichment analysis.

### Follow-up of miRNA association results—regional association plots

A window-based approach that included common variants in miRNAs and flanking sequences was applied. To determine whether the signal was associated with any of the miRNAs of interest, visual inspection of the regional association plots was performed.

Regional association results from our BD GWAS^6^ were plotted for all associated miRNAs and their ±500-kb flanking regions using LocusZoom.^36^ A signal was considered miRNA-associated if the top SNP of the region was located at, or was in high or moderate LD (*r*^2^>0.6) with, the miRNA locus.

### Follow-up of miRNA association results—miRNA brain expression

To investigate expression of the associated miRNAs in the human brain, data from a recent study of miRNA expression patterns in the developing human brain were re-analyzed.^37^ A miRNA was defined as showing brain expression if it had a total read count of >120 across all investigated samples.^37^

In addition, miRNA expression was measured in rat cortical neurons and forebrain. All procedures involving animals followed the guidelines of the German Animal Protection Legislation and the experiments were approved by the Local Committee for Animal Health (RP Gießen). Total RNA was isolated from the postnatal day 15 rat forebrain or synaptosomes, as described elsewhere.^38^ Briefly, the total RNA from the forebrain of postnatal day-15 Sprague–Dawley rat pups was extracted using peqGOLD TriFast reagent (Peqlab, Erlangen, Germany) in accordance with the manufacturer's instructions. Small RNA libraries were constructed and sequenced at the EMBL genomic core facility (Heidelberg, Germany) using the HiSeq platform (Illumina, San Diego, CA, USA). The web-based software MiRanalyzer was used to determine miRNA expression levels (http://bioinfo2.ugr.es/miRanalyzer/miRanalyzer.php.).^39^

### miRNA target gene analysis

Targets of the associated miRNAs 499, 708 and 1908 were obtained from TargetScan (Release 6.2).^40^ The Allen human brain atlas (http://www.brain-map.org/)^41^ was consulted to determine whether predicted target genes are expressed in the human brain. Target genes were considered brain-expressed if they had shown expression in the hippocampal formation in at least four of the six donor brains. Gene-based *P*-values for all brain-expressed miRNA targets were calculated using versatile gene-based test for GWAS,^34^ and our BD GWAS data set.^6^ To capture regulatory regions, the default settings in versatile gene-based test for GWAS were used. Enrichment of associated targets was calculated as follows: the number of associated target genes for each miRNA was compared with the number of associated genes from 100 000 random target sets of brain-expressed genes. Each target gene set comprised the same number of genes as the miRNA target genes itself.

### Pathway analysis of target genes

The subsequent analyses were restricted to brain-expressed target genes of *miR-499*, *miR-708* and *miR-1908*, with a gene-based association *P*-value of <0.05. If the chromosomal distance between two target genes was below 100 kb or if the top SNPs of two target genes were in strong or moderate LD (*D*'>0.4), only the target gene with the lowest gene-based *P*-value was retained in the pathway analysis to ensure the independency of association signals. In total, 107 target genes were included in the pathway analyses (Supplementary Box 1). Gene ontology (GO) and Kyoto Encyclopaedia of Genes and Genomes pathway testing was performed using the WebGestalt (Web-based Gene Set Analysis Toolkit) for the brain-expressed, BD-associated target genes of the three associated miRNAs. Bonferroni correction was used to adjust for multiple testings. Significant pathways were filtered to achieve a minimum of three genes per set.

### Functional analyses of miR-499 and miR-708 in rat hippocampal neurons

To test the possible involvement of *miR-499* or *miR-708* in the regulation of synaptic function, experiments were performed to investigate the effect of *miR-499* and *miR-708* overexpression on dendritic spine morphogenesis in primary rat hippocampal neurons. We initially focused on overexpression, as this can be easily achieved by the transfection of expression plasmids containing pri-miRNA cassettes. miRNA overexpression constructs were generated by inserting the respective pri-miRNA sequences into the 3'-untranslated repeat of the luciferase reporter gene within pmiRGLO (Promega, Madison, WI, USA). Thereby, luciferase reporter assays could be used to monitor the efficiency of pri-miRNA processing. To investigate the potential involvement of *miR-499-5p* and *miR-708-5p* in dendritic spine morphogenesis, hippocampal neurons of embryonic day-18 Sprague–Dawley rats (Charles River Laboratories, Sulzfeld, Germany) were transfected with miRNA-overexpressing constructs for 6 days before fixation. Images with a resolution of 1024 × 1024 pixels were obtained using a LSM5 Zeiss Pascal confocal microscope (Jena, Germany) and in a magnification of × 63 /1.4. A maximum projection was reconstructed with the Zeiss LSM 510 Meta software from a z-stack consisting of seven optical slices at 0.45-μm interval. The average intensity of an area of 2180 nm^2^ containing 250–300 spines per cell was measured using the ImageJ 1.48v software (National Institutes of Health, Bethesda, MD, USA), as described elsewhere.^38^ During imaging and analysis, the investigator was blind to the transfection condition.

## Results

**Figure 1:**
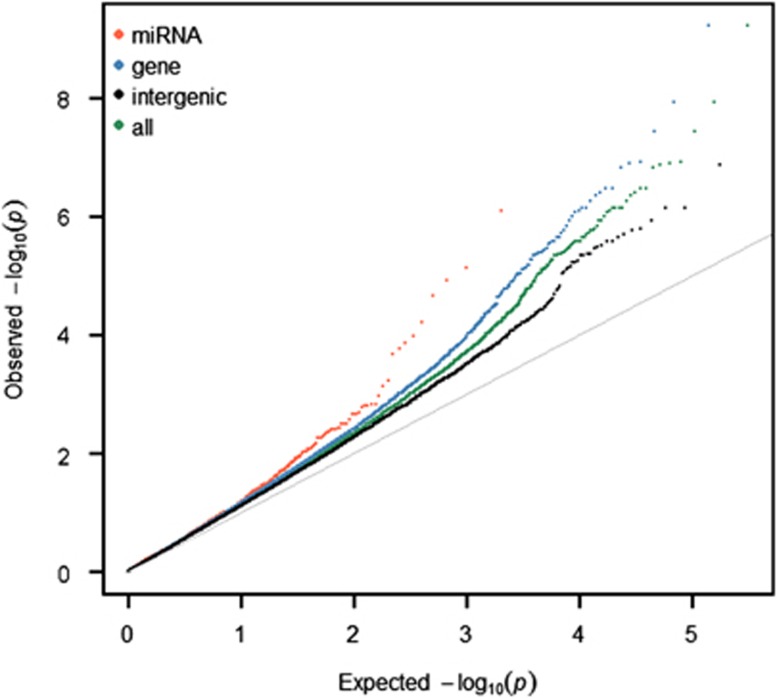
Quantile–quantile (Q–Q) plot of single-nucleotide polymorphism (SNP) *P*-values. The −log10 of the observed genome-wide association studies (GWAS) *P*-values for linkage disequilibrium (LD)-pruned SNPs (on the *y* axis) are plotted versus the −log 10 of the expected *P*-values (under null, on the *x* axis). The solid line represents expected uniform distribution. Red dots represent the data distribution of *P*-values of SNPs at microRNA loci; blue dots represent SNPs in genes; black dots represent *P*-values of intergenic SNPs; and green dots represent the data distribution of all SNPs.

Overall, the nominal *P*-values of SNPs at miRNA loci were enriched with lower values than would be expected with a uniform *P*-value distribution (Figure 1). This deviation from the expected normal Q–Q plot distribution indicates a general enrichment for miRNAs among BD-associated SNPs. Category testing for different *P*-value thresholds revealed a significant enrichment for BD-associated SNPs in miRNA loci for *P*-values<1 × 10^−4^ (Supplementary Table 1). This deviation was also observed among SNPs in genes but not for intergenic SNPs.

Gene-based analysis in our BD GWAS data^6^ generated nominally significant *P*-values for 98 of the 609 miRNAs. These included *miR-2113*, which was located at the genome-wide significant locus on chromosome 6q16.1 in the original BD GWAS analyses.^6^ After correction for multiple testing, nine miRNAs showed a significant association with BD (Table 1). The additional calculation of gene-based tests for these nine miRNAs on the basis of 1000 Genomes LD structure generated nominal *P*-values of ⩽7.20 × 10^−5^ (Supplementary Table 2).

**Figure 2:**
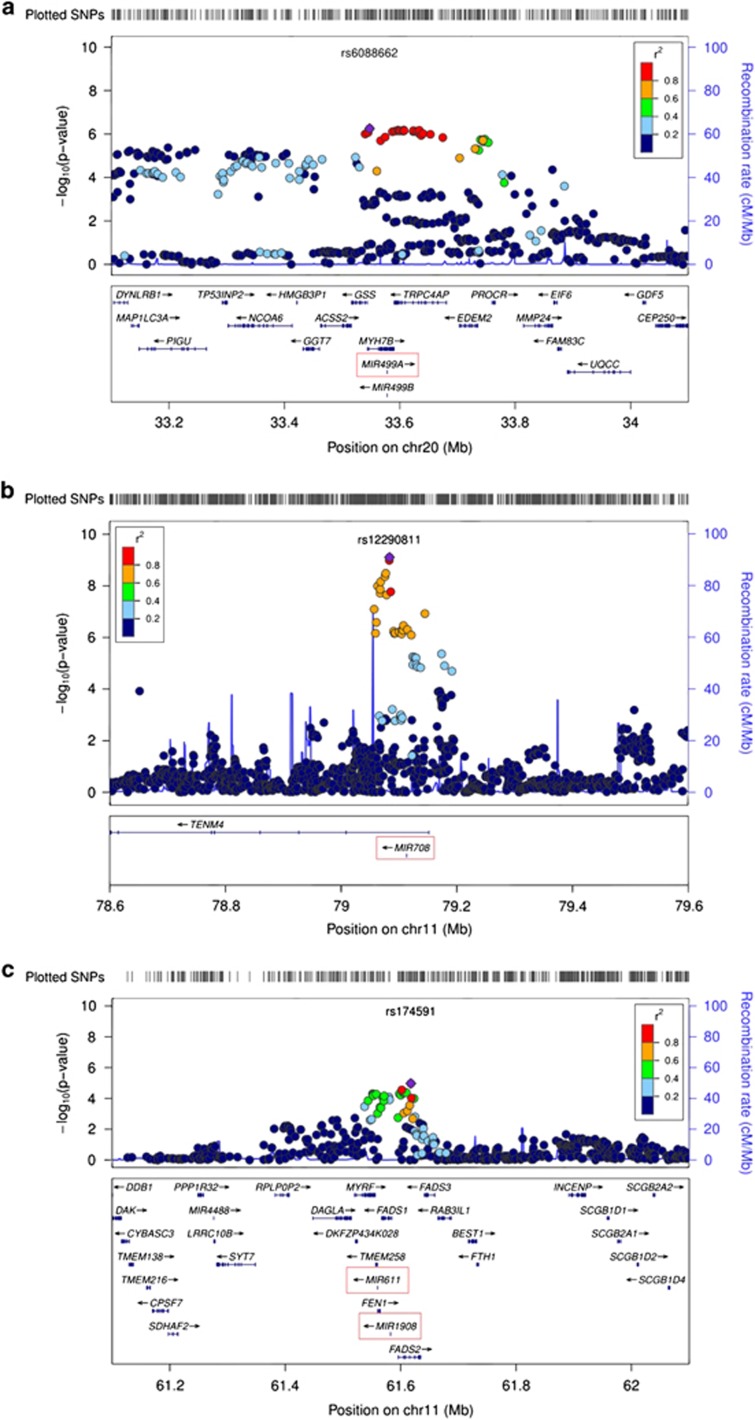
Regional association plots of *miR-499*, *miR-708* and *miR-1908*. Regional association results for the three most promising associated microRNAs *miR-499* (**a**), *miR-708* (**b**) and *miR-1908* (**c**), and their ±500-kb flanking regions were plotted using LocusZoom (Pruim *et al.*^36^). The plot of *miR-1908* (**c**) includes *miR-611*, which is also localized at the depicted chromosomal locus.

Visual inspection of the regional association plots revealed a miRNA-associated signal for five of the nine miRNAs (Figure 2, Supplementary Figures 1–4).

The re-analysis of the expression data from Ziats and Rennert^37^ revealed that five of the nine miRNAs were expressed in the human brain (Table 1).

Three of these (*miR-499, miR-708* and *miR-135a-1*) were also found to be expressed in the rat forebrain. This method could not be used to investigate the expression of the other miRNAs, as they are not expressed in rats.^35^

The regional association plots and the miRNA expression data in human brain tissue suggest that the three brain-expressed miRNAs, that is, *miR-499*, *miR-708* and *miR-1908*, are the most promising candidates for further analyses. The three miRNAs had 296, 181 and 67 target genes, respectively. Of these 286, 174 and 56 showed brain expression (Table 2).

The target gene enrichment analysis showed no significant enrichment of BD-associated genes within the targets of *miR-499*, *miR-708* or *miR-1908* (Table 2). After Bonferroni correction, *miR-1908* had one (*KLC2*) and *miR-708* had two significant target genes (*NRAS* and *CREB1*), whereas *miR-499* had four significant target genes (*GPC6, C16orf72, WDR82* and *CACNB2*).

Pathway testing revealed 18 significant canonical pathways that are driven by brain-expressed target genes of the three miRNAs (Table 2). For each miRNA, the results of the GO analysis are presented as directed acyclic graphs (Supplementary Figure 5). The target genes that drive a particular pathway are listed in Supplementary Table 3.

**Figure 3:**
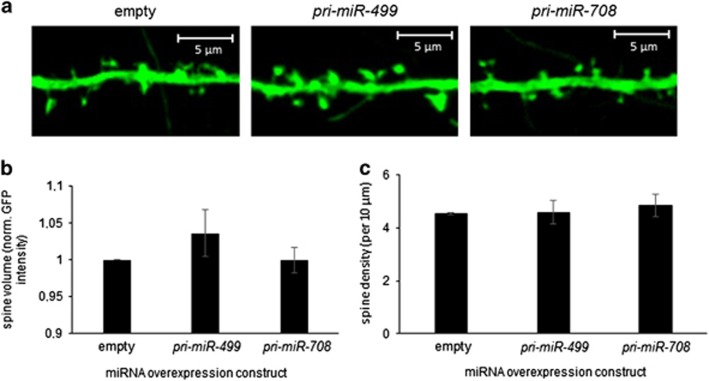
Effect of the overexpression of *miR-499* and *miR-708* on dendritic spine size and density in primary rat hippocampal neurons. DIV14 primary hippocampal neurons were transfected with: (i) empty pmirGLO (250 ng) or (ii) pmirGLO (250 ng) containing *pri-miR-499* or *pri-miR-708* in the 3'-untranslated repeat of the Firefly luciferase gene and green fluorescent protein (GFP). The transfected neurons were then cultured until DIV19 and fixed for fluorescence microscopy. (**a**) Representative images for cells transfected with the indicated pmirGLO constructs or GFP only. A three-dimensional reconstruction was made from seven 45-μm stacks; scale bars, 5 μm. (**b**) Spine volume quantification of hippocampal neurons transfected with the indicated pmirGLO constructs. Values are represented as means±s.d. (*n*=3; 24 neurons per condition with a 200–250 spine count per cell). (**c**) Spine density of hippocampal neurons transfected with the indicated pmirGLO constructs. Values are represented as means±s.d. per 10 μm dendritic length (*n*=3; 24 neurons per condition). Data are presented as the mean of three independent transfections normalized to the empty pmirGLO condition±s.d.

Luciferase assays revealed efficient processing of *pri-miR-499*, but not *pri-miR-708*, upon transfection of the respective constructs in neurons (Supplementary Figure 6). Overexpression *of miR-499* led to a small and statistically nonsignificant increase in spine volume (Figure 3), but no effect on spine density was observed. As expected, transfection of the non-effective *miR-708* expression construct had no significant effect on spine morphological parameters. Taken together, these results suggest that increasing levels of the BD-associated *miR-499* have no—or only minimal—modulatory function during dendritic spine morphogenesis.

## Discussion

The present genetic association results for miRNA-coding genes suggest that miRNAs and their target genes may be implicated in the development of BD. The nominal *P*-values of SNPs at miRNA loci showed early deviation from the expected null line in the Q–Q plot, and this leftward shift reflects an enrichment of BD-associated SNPs at miRNA loci.

For the nine miRNAs that withstood Bonferroni correction, we additionally calculated the gene-based tests on the basis of the 1000 Genomes LD structure. This analysis revealed nominally gene-based *P*-values ⩽7.20 × 10^−5^ for all nine miRNAs, indicating that the results of gene-based tests on the basis of either HapMap phase 2 or 1000 Genomes Project data are highly comparable using our BD GWAS data.

Eight of the nine associated miRNAs were located in a host gene, including the three brain-expressed miRNAs *miR-499*, *miR-708* and *miR-1908*. Recent studies have reported a high correlation between the expression of a host gene and the resident miRNA.^15, 42^ Previous authors have hypothesized that this finding may be because of the fact that miRNAs residing in introns are likely to share their regulatory elements and primary transcript with their host gene.^24^ Some authors point out that host genes and their resident miRNAs may even have synergistic effects, which would have important implications for the fine-tuning of gene expression patterns in the genome.^43, 44^ On the basis of the present genetic association results, it is impossible to determine whether the association was attributable to the host gene, the miRNA or both. Further analyses are therefore warranted to clarify this, which was beyond the scope of the present analysis. However, the general enrichment of BD-associated SNPs at miRNA loci (Figure 1) and the results of our target gene analyses support the hypothesis that the majority of the associated miRNAs are implicated in BD etiology.

Regional association plots and expression data suggest that the miRNAs *miR-499*, *miR-708* and *miR-1908* are the most promising candidates in terms of the development of BD.

The miRNA *miR-499* is located in a region on chromosome 20q11 that showed genome-wide significant association in a previous GWAS of BD.^45^ As *miR-499* is located in a region of high LD, which includes the genes *GSS*, *MYH7B* and *TRPC4AP* (Figure 2), further analyses of this chromosomal region are required to refine the association signal.^45^ However, *miR-499* represents a very promising candidate in this region.

*MiR-499* regulates apoptotic pathways involving the calcium-dependent protein phosphatase calcineurin.^46^ A recent study demonstrated an upregulation of *miR-499* in the prefrontal cortex of patients with depression.^47^ In a study of exosomal miRNA expression, *miR-499* showed differential expression in the post-mortem brains of BD patients compared with controls.^48^ When considering a possible pathomechanism, it is important to note that a common SNP (rs3746444) is located in the seed region of the mature *miR-499-3p*.^49^ This seed region is crucial for both the recognition of the target sites and the binding of the target genes. The SNP rs3746444 was not among the 2 267 487 SNPs analyzed in our large BD meta-analysis.^6^ However, rs3746444 achieved a nominally significant *P*-value of 0.0023 (risk allele: rs3746444-G) in a combined analysis of the seven MooDS samples (2266 patients and 5028 controls),^6^ which excluded the PGC data set.^3^ Furthermore, the allele rs3746444-G has been associated with hallucinations and lack of motivation in schizophrenia patients.^50^ This suggests that this SNP may confer susceptibility to BD by influencing depressive and psychotic endophenotypes. However, it may only partly explain the association signal at this locus.

Our target gene analysis revealed that *miR-499* had four significant target genes, including the previously reported genome-wide significant risk gene for psychiatric disorders *CACNB2*.^51^

Brain-expressed target genes of *miR-499-5p* exhibited an enrichment in biological processes related to cerebral development, which might however, at least partly, reflect the fact that our pathway analysis was restricted to brain-expressed genes. In addition, our pathway analysis indicates a potential role of *miR-499* in the regulation of the actin cytoskeleton. Interestingly, this pathway has been identified in a previous investigation of differentially and concordantly expressed genes enriched in association signals for schizophrenia and BD.^52^ Substantial research evidence suggests that the rearrangement of the cytoskeleton is crucial for neuronal cell migration and maturation, neurite outgrowth and maintenance of synaptic density and plasticity.^53, 54, 55, 56^ These combined data suggest that *miR-499* is an interesting candidate for BD pathogenesis.

The miRNA *miR-708* is located in the first intron of *ODZ4* (odd Oz/ten-m homolog 4, *TENM4*), which has been reported as a genome-wide significant susceptibility gene for BD.^3^

A recent study of postpartum psychosis—a disorder that often heralds the incipient onset of BD^57^—suggested differential expression of *miR-708* in the monocytes of affected patients compared with controls.^58^ In another study, Xu *et al.*^59^ demonstrated an altered expression profile for *miR-708* in mouse hippocampal neurons and showed that this was mediated by oxidative stress. Another recent study found that *miR-708* regulated the expression of neuronatin, which is a membrane protein in the endoplasmic reticulum. Interestingly, the neuronatin-mediated regulation of intracellular Ca^2+^ levels has been implicated in cell migration and neural induction within embryonic stem cells.^60^

Our target gene analysis revealed that *miR-708* had two significant target genes. These include *CREB1* that has previously been identified as a susceptibility gene for major depressive disorder.^61, 62, 63^ In addition, *CREB1* was found to be associated with BD in a recent study of large-scale BD samples^64^ that included 8403 patients and 11 588 controls of our BD GWAS.^6^ However, the present pathway analysis provided no strong evidence for an enrichment of biological processes of relevance to psychiatric disorder.

*MiR-1908* is located in the first intron of the fatty acid desaturase 1 (*FADS1*) gene on chromosome 11. To date, few published studies have investigated the function of *miR-1908*. One recent study implicated *miR-1908* as a cancer biomarker.^65^ A further study found that *miR-1908* belonged to a miRNA cluster that downregulates the MARK1 signaling pathway, thus altering cell proliferation and differentiation.^66^

Pathway analysis results for *miR-1908* indicate a potential role of the miRNA-regulated target gene network in key neuronal processes (GO subcategories: neuron projection and nervous system development). As these pathways showed the strongest enrichment, further research into *miR-1908* and its regulated network appears to be warranted.

Although initial efforts have been made to elucidate the regulation of miRNA expression,^67^ the manner in which miRNA expression and processing are regulated remains largely unknown. Given that pri-miRNAs have a length of 100–1000 bp,^16^ the present study investigated common variants at the miRNA loci and ±20 kb flanking sequences in order to capture possible regulatory regions. However, further analyses of the regulation of miRNA expression by common variants are required to determine whether, and how, the presently described association signals influence the expression levels and function of the implicated miRNAs. The present approach did not allow investigation of SNPs with *trans*-expression quantitative trait loci (eQTL) effects on miRNAs. As recent studies suggest that ~50% of the identified miRNA eQTLs are *trans*-eQTLs,^68^ investigations into the association between miRNA *trans*-eQTLs and BD are indicated.

The results of the functional analyses of *miR-499* and *miR-708* in rat hippocampal neurons revealed no major contribution of these miRNAs to the morphogenesis of dendritic spines, which represent the major sites of synaptic contact. However, only the results for *miR-499* can be considered robust, as the *miR-708* expression construct did not increase *miR-708* in primary neurons effectively. Alternative strategies for *miR-708* expression, together with *miR-499/708* loss-of-function approaches, must be tested before definite conclusions regarding the role of these miRNAs in dendritic spine morphogenesis can be drawn. Moreover, to obtain more comprehensive insights into the potential effects of these miRNAs on synaptic function, future experiments should be complemented by immunocytochemistry analyses of synaptic marker proteins and electrophysiological recordings. Beyond a potential involvement in dendritic spine morphogenesis, these miRNAs could also regulate other aspects of neuronal morphology, such as dendrite arborization or axon growth, which could be tested in future studies.

## Conclusion

The results of the present miRNA and target gene analyses suggest that the brain-expressed miRNAs *miR-499*, *miR-708* and *miR-1908* may contribute to the development of BD. Further research is warranted to elucidate the involvement of these miRNAs and their downstream pathways in BD.

## Figures and Tables

**Table 1:** Results of the gene-based tests for the nine microRNAs that withstood Bonferroni correction

*miRNA*	*Chr*	*nSNPs*	*Top SNP*	*p Top SNP*	*p Corr Gene*	*miRNA-assoc. signal*	*Expr. hum. brain*
*miR-499*	20	27	rs3818253	6.58 × 10^−7^	0.0012	Yes	Yes
*miR-640*	19	21	rs2965184	7.23 × 10^−7^	0.0012	Yes	No
*miR-708*	11	72	rs7108878	3.45 × 10^−7^	0.0012	Yes	Yes
*miR-581*	5	36	rs697112	3.61 × 10^−6^	0.0073	Yes	No
*miR-644*	20	12	rs7269526	1.22 × 10^−5^	0.0104	No	No
*miR-135a-1*	3	20	rs9311474	2.16 × 10^−5^	0.0122	No	Yes
*let-7 g*	3	9	rs6445358	2.23 × 10^−5^	0.0305	No	Yes
*miR-1908*	11	16	rs174575	2.85 × 10^−5^	0.0353	Yes	Yes
*miR-611*	11	23	rs174535	5.03 × 10^−5^	0.0457	No	No

Abbreviations: Chr, chromosome; expr. hum. brain, expression in the human brain according to Ziats and Rennert;^37^ miRNA, microRNA; miRNA-assoc. signal, specificity of the associated finding in the regional association plot; p Corr Gene, Bonferroni-corrected gene-based *P*-value; p Top SNP, *P*-value of the Top SNP within gene; nSNPs, number of investigated SNPs; SNP, single-nucleotide polymorphism.

**Table 2:** Target gene and pathway analysis for *miR-499*, *miR-708* and *miR-1908*

*MicroRNA*	*No. of brain-expressed target genes*	*No. of brain-expressed target genes*, P*<0.05*	*P enrichment*	*No. of significant targets (corr)*	*No. of significant pathways*
*miR-499-5p*	286	59	0.7172	4	12
*miR-708-5p*	174	37	0.9265	2	1
*miR-1908-5p*	56	17	0.1422	1	5

Abbreviations: No. of significant pathways, number of significant pathways at *P*≤0.05; No. of significant targets (corr), number of significant target genes after Bonferroni correction for multiple testing; P enrichment*, P*-value of the enrichment analysis (*X*^2^-test).

Results of the target gene analysis for the three brain-expressed microRNAs *miR-499*, *miR-708* and *miR-1908* that were associated with bipolar disorder after correction for multiple testing.
