# Supplementary material for: Genome-wide analysis implicates microRNAs and their target genes in the development of bipolar disorder
Source: Transl Psychiatry. 2015 Nov 10;5(11):e678–. doi: 10.1038/tp.2015.159 (PMC5068755; doi:10.1038/tp.2015.159)
Supplement: Supplementary Information [file tp2015159x1.docx]

Supplementary information: Forstner, Hofmann *et al.*, Genome-wide analysis implicates microRNAs and their target genes in the development of bipolar disorder

**Table of contents**

[**Supplementary Figure 1:** Regional association plot of *miR-640* 2](#_Toc412107450)

[**Supplementary Figure 2:** Regional association plot of *miR-581* 3](#_Toc412107451)

[**Supplementary Figure 3:** Regional association plot of *miR-644* and *miR-499* 4](#_Toc412107452)

[**Supplementary Figure 4:** Regional association plot of *let-7g and miR-135a-1* 5](#_Toc412107453)

[**Legend:** Supplementary Figures 1-4 6](#_Toc412107457)

[**Supplementary Figure 5:** Directed Acyclic Graphs (DAG) of the enriched GO categories 7](#_Toc412107458)

[**Supplementary Figure 6:** Validation of *miR-499* and *miR-708* processing using a dual-Luciferase reporter assay in rat hippocampal neurons 9](#_Toc412107459)

[**Supplementary Table 1:** Subcategory enrichment for different p value thresholds 10](#_Toc412107459)

[**Supplementary Table 2:** Comparison of the results of the gene-based tests based on different LD structure 11](#_Toc412107460)

[**Supplementary Table 3:** Significant biological pathways in microRNA target gene data sets 12](#_Toc412107460)

[**Supplementary Box 1**: List of the 107 brain-expressed microRNA target genes associated with bipolar disorder at gene-based p < 0.05 13](#_Toc412107461)

## Supplementary Figure 1: Regional association plot of *miR-640*


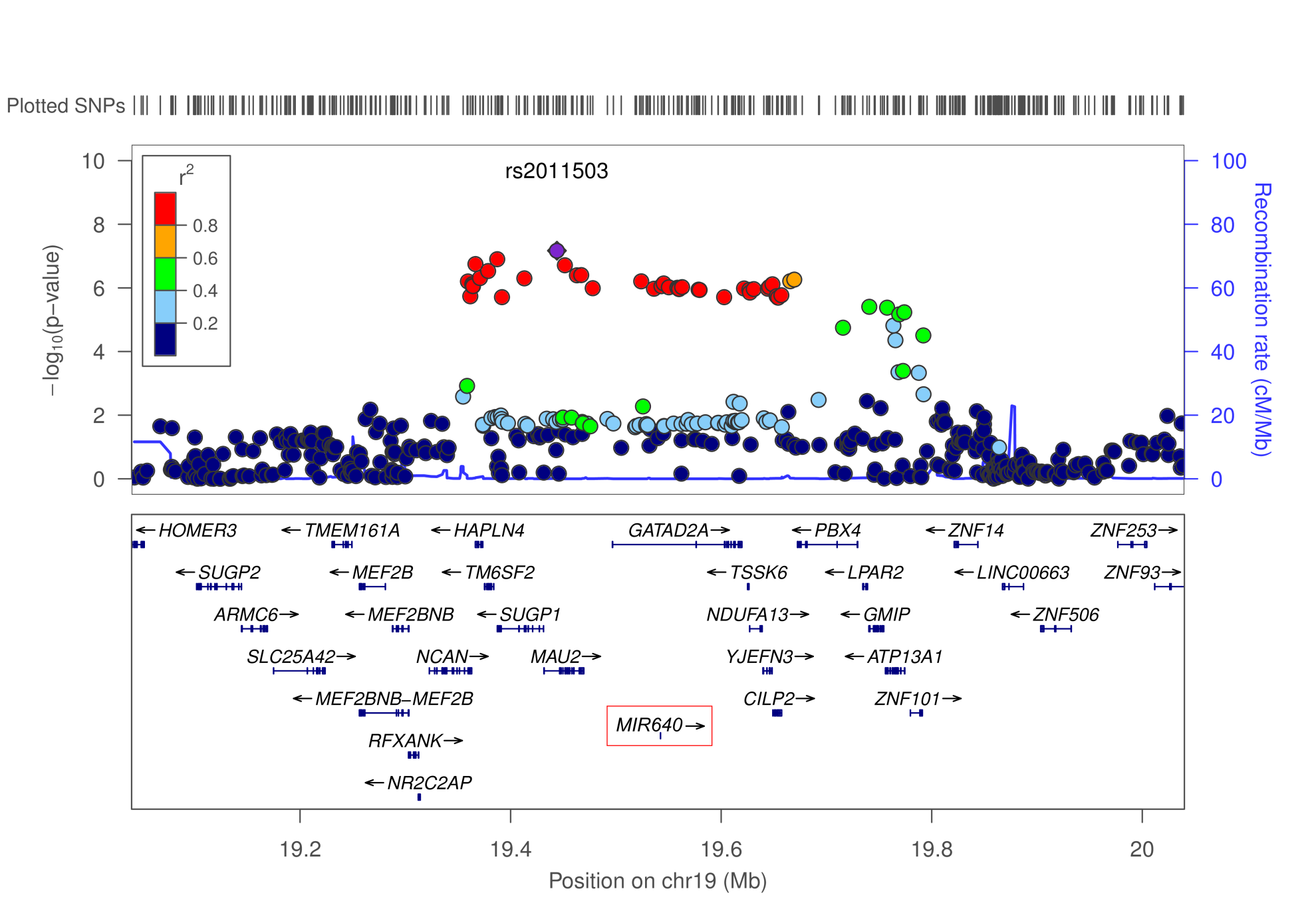


## Supplementary Figure 2: Regional association plot of *miR-581*


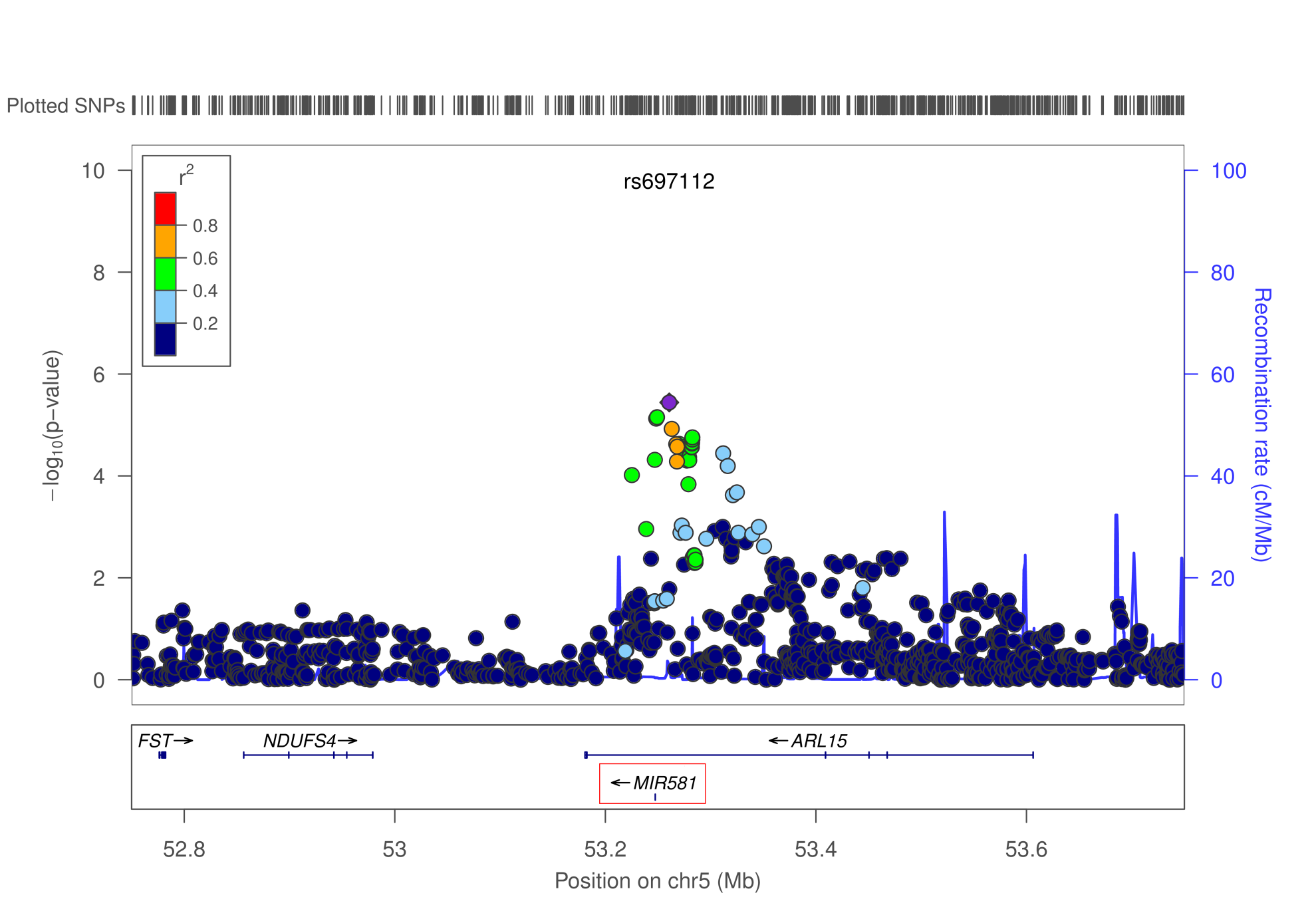


Supplementary Figure 3: Regional association plot of *miR-644* and *miR-499*


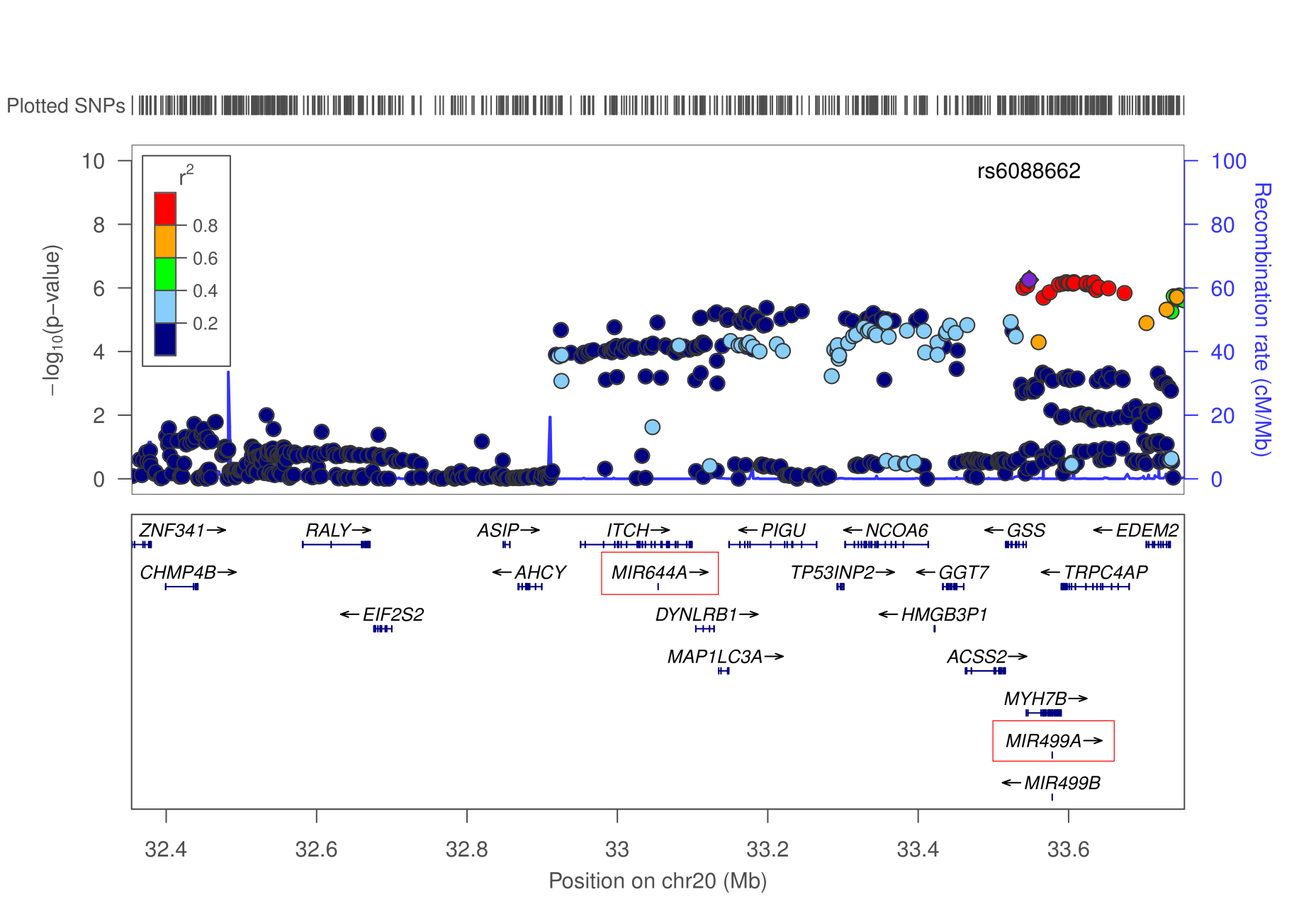


Supplementary Figure 4: Regional association plot of *let-7g* and *miR-135a-1*


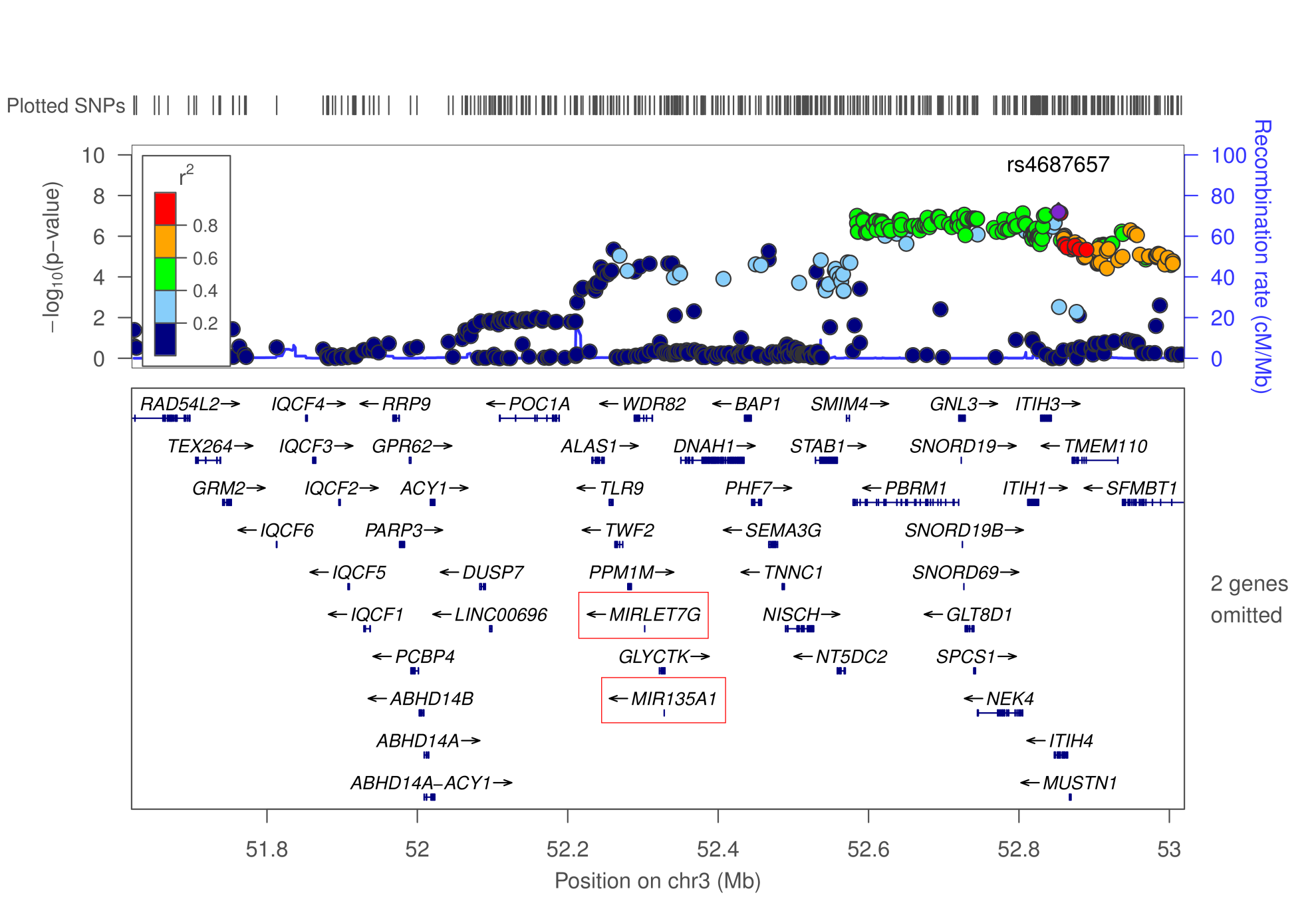


Legend Supplementary Figures 1-4**:** Regional association results for all associated miRNAs and their +/- 500 kb flanking regions were plotted using LocusZoom (Pruim et al., 2010). A signal was considered miRNA-associated if the Top SNP of the region was located at the miRNA locus or if it was in high or moderate LD (r^2^>0.6) with the miRNA locus.

## Supplementary Figure 5: Directed Acyclic Graphs (DAG) of the enriched GO categories


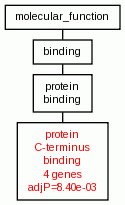
**
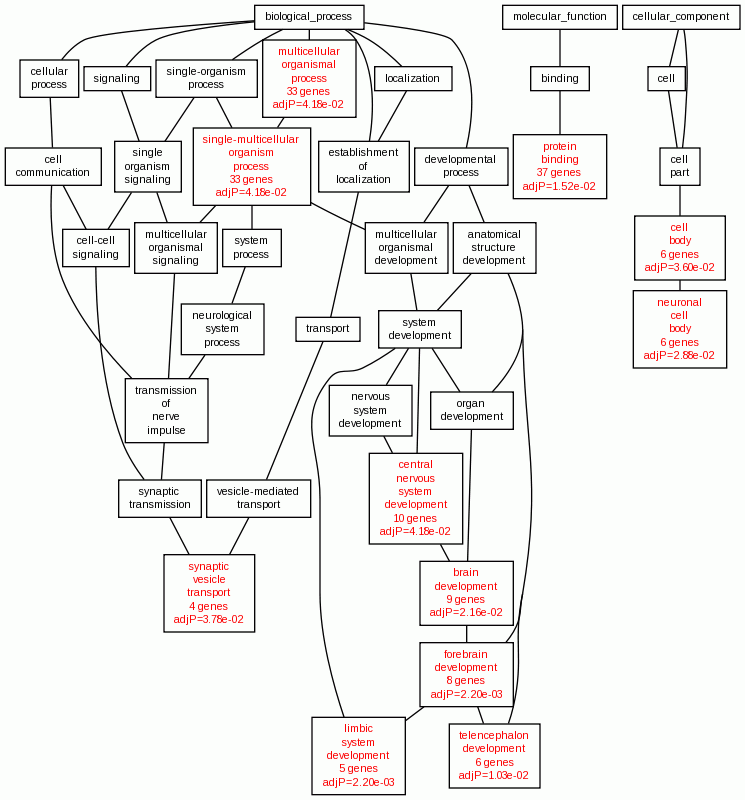
A B**

**C**


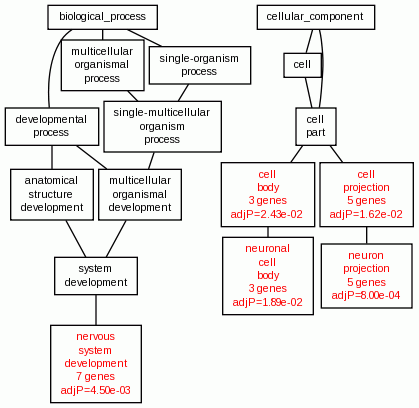


**Legend Supplementary Figure 5**: Target gene sets of the three microRNAs 499, 708 and 1908 were subjected to gene ontology (GO) analysis. For each microRNA the results of the GO analysis are presented as directed acyclic graphs. Significant results (p < 0.05, Bonferroni corrected) for pathways which contained at least three target genes are shown in red. **A** *miR-499* **B** *miR-708* and **C** *miR-1908*.

**Supplementary Figure 6: Validation of *miR-499* and *miR-708* processing using a dual-Luciferase reporter assay in rat hippocampal neurons**


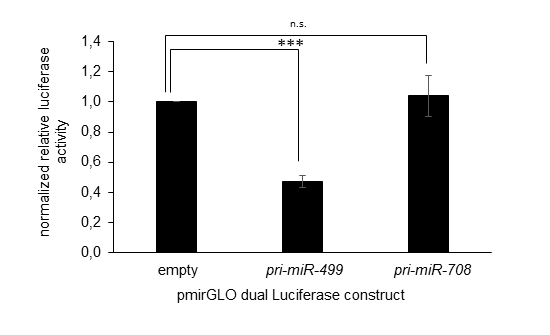


## Legend Supplementary Figure 6:

Relative Luciferase activity (RLA) of DIV 10 primary rat hippocampal neurons transfected with the pmirGLO dual Luciferase reporter construct containing indicated primary-miRNA genes in the Firefly 3’UTR. Processing of the primary-miRNA genes leads to a loss of the protective poly-A tail, resulting in a fast degradation of the Firefly mRNA and therefore to reduced luciferase activity. Data are presented as the mean of three independent experiments normalized to the empty reporter construct ± standard deviation; n.s. = non significant *p>0.05 **p>0.01 ***p<0.005 (t-test).

## Supplementary Table 1: Subcategory enrichment for different p value thresholds

| **p value threshold** | **SNPs in miRNA +/- 20 kb** | **SNPs in genes** | **Intergenic SNPs** |
| --- | --- | --- | --- |
| <1 x 10^-6^ | 0.11 | 4.00 x 10^-4^ | > 0.99 |
| ≥1 x 10^-6^ & <1 x 10^-4^ | 0.037 | 2.39 x 10^-7^ | > 0.99 |
| ≥1 x 10^-4^ & <0.05 | 0.041 | 1.22 x 10^-15^ | > 0.99 |
| ≥0.05 | 0.98 | > 0.99 | 5.53 x 10^-18^ |

**Legend Supplementary Table 1:** Subcategory testing for different BD-association p value thresholds for the categories SNPs in miRNA loci (+/- 20 kb), SNPs in genes, intergenic SNPs. Enrichment was calculated using a one-sided fisher’s exact test.

**Supplementary Table 2: Comparison of the results of the gene-based tests for the nine microRNAs that withstood Bonferroni correction based on the LD structure derived from HapMap phase 2 or the 1,000 Genomes Project**

| **MicroRNA** | **Chr** | **Top SNP** | **p Top SNP** | **nSNPs HM2** | **p Gene HM2** | **nSNPs 1000G** | **p Gene 1000G** |
| --- | --- | --- | --- | --- | --- | --- | --- |
| *miR-499* | 20 | rs3818253 | 6.58 x 10^-7^ | 27 | 2.00 x 10^-6^ | 27 | 1.00 x 10^-6^ |
| *miR-640* | 19 | rs2965184 | 7.23 x 10^-7^ | 21 | 2.00 x 10^-6^ | 21 | 1.00 x 10^-6^ |
| *miR-708* | 11 | rs7108878 | 3.45 x 10^-7^ | 72 | 2.00 x 10^-6^ | 72 | 3.00 x 10^-6^ |
| *miR-581* | 5 | rs697112 | 3.61 x 10^-6^ | 36 | 1.20 x 10^-5^ | 34 | 1.40 x 10^-5^ |
| *miR-644* | 20 | rs7269526 | 1.22 x 10^-5^ | 12 | 1.70 x 10^-5^ | 12 | 8.00 x 10^-6^ |
| *miR-135a-1* | 3 | rs9311474 | 2.16 x 10^-5^ | 20 | 2.00 x 10^-5^ | 20 | 2.00 x 10^-5^ |
| *let-7g* | 3 | rs6445358 | 2.23 x 10^-5^ | 9 | 5.00 x 10^-5^ | 10 | 2.50 x 10^-5^ |
| *miR-1908* | 11 | rs174575 | 2.85 x 10^-5^ | 16 | 5.80 x 10^-5^ | 16 | 6.10 x 10^-5^ |
| *miR-611* | 11 | rs174535 | 5.03 x 10^-5^ | 23 | 7.50 x 10^-5^ | 23 | 7.20 x 10^-5^ |

**Legend Supplementary Table 2:** Abbreviations: Chr = Chromosome; p Top SNP = p value of the Top SNP within gene; nSNPs = number of investigated SNPs; HM2 = based on HapMap phase 2 data, 1000G = based on the 1,000 Genomes Project data, p Gene = nominal gene-based p value.

## Supplementary Table 3: Significant biological pathways in microRNA target gene data sets

| **microRNA** | **Type** | **Pathway** | | **no. genes in subcategory** | **no. target genes** | **p corr.** | **gene symbol** |
| --- | --- | --- | --- | --- | --- | --- | --- |
| ***miR-499*** | KEGG | Regulation of actin cytoskeleton | | 213 | 3 | 0.0032 | *ENAH, VAV3, PFN2* |
|  | GO | forebrain development | | 281 | 8 | 0.0022 | *LMX1A, BCL11B, EPHA5, CNTNAP2, SLC8A3, ETS1, HOOK3, ZEB2* |
|  | GO | limbic system development | | 73 | 5 | 0.0022 | *LMX1A, EPHA5, CNTNAP2, ETS1, ZEB2* |
|  | GO | telencephalon development | | 168 | 6 | 0.0103 | *LMX1A, BCL11B, EPHA5, CNTNAP2, SLC8A3, ZEB2* |
|  | GO | protein binding | | 7337 | 37 | 0.0152 | *AAK1, H2AFZ, ENAH, CNTNAP2, VPS13A, PFN2, ETS1, AP3S1, EEA1, RIMS1, CACNB2, QKI, PIM1, MARCKS, TBC1D15, CPSF6, TOP1, VAV3, CHD9, SLC8A3, DYNLT1, HOOK3, HNRNPC, ILF3, PTCH1, EFHC1, WDR82, UHRF1BP1, GPC6, KCNN3, PURB, FKBP5, SOX5, PTPN14, SPAST, PRKAR1A, ZEB2* |
|  | GO | brain development | | 502 | 9 | 0.0216 | *BCL11B, SLC8A3, HOOK3, PTCH1, LMX1A, EPHA5, CNTNAP2, ETS1, ZEB2* |
|  | GO | neuronal cell body | | 291 | 6 | 0.0288 | *EPHA5, EFHC1, CNTNAP2, TANC1, SLC8A3, TOP1* |
|  | GO | cell body | | 312 | 6 | 0.0360 |  |
|  | GO | synaptic vesicle transport | | 68 | 4 | 0.0378 | *RIMS1, AP3S1, PFN2, EEA1* |
|  | GO | central nervous system development | | 688 | 10 | 0.0418 | *BCL11B, SLC8A3, HOOK3, PTCH1, LMX1A, EPHA5, CNTNAP2, ETS1, SOX5, ZEB2* |
|  | GO | multicellular organismal process | | 5644 | 33 | 0.0418 | *BCL11B, ENAH, CNTNAP2, VPS13A, PFN2, ETS1, KCNQ5, AP3S1, EEA1, RIMS1, CACNB2, QKI, PIM1, TOP1, VAV3, SLC8A3, TMEM2, DYNLT1, JPH1, HOOK3, PTCH1, EPHA5, TANC1, KCNN3, LMX1A, PURB, PTPN14, SPAST, SOX5, ZEB2, PRKAR1A, RNF114, ROD1/PTBP3* |
|  | GO | single-multicellular organism process | | 5612 | 33 | 0.0418 |  |
|  | | |  |  |  |  |  |
| ***miR-708*** | GO | protein C-terminus binding | | 158 | 4 | 0.0084 | *ATXN1, SHANK3, PFKM, FOXN3* |
|  |  |  | |  |  |  |  |
| ***miR-1908*** | GO | neuron projection | | 651 | 5 | 0.0008 | *MINK1, SLC12A5, SLC17A7, NCDN, KLC2* |
|  | GO | nervous system development | | 1724 | 7 | 0.0045 | *MINK1, PCDHA1, MDGA1, OTX1, GDF11, NRGN, NCDN* |
|  | GO | cell projection | | 1230 | 5 | 0.0162 | *MINK1, SLC12A5, SLC17A7, NCDN, KLC2* |
|  | GO | neuronal cell body | | 291 | 3 | 0.0189 | *SLC12A5, NCDN, EEF1A2* |
|  | GO | cell body | | 312 | 3 | 0.0243 |  |

**Legend Supplementary Table 3:** Significant pathways with a minimum of three genes included per set are depicted in Supplementary Table 3. Abbreviations: KEGG = Kyoto Encyclopedia of Genes and Genomes; GO = Gene Ontology; no. genes in subcategory = number of reference genes in KEGG/GO subcategory; no. target genes = number of microRNA target genes contained in subcategory; p corr = Bonferroni corrected p value.

##

## Supplementary Box 1: List of the 107 brain-expressed microRNA target genes associated with bipolar disorder at gene-based p < 0.05

***miR-499-5p*:**

*GPC6, C16orf72, WDR82, CACNB2, BCL11B, HNRNPC, EFHC1, ILF3, SLC8A3, QKI, ANKRD40, MARCKS, DYNLT1, AAK1, PTCH1, PRKAR1A, EPHA5, TANC1, VAV3, SOX5, MTX3, CPSF6, FKBP5, LHFPL2, RIMS1, RBMS3, TMEM2, RRP1B, RSBN1, UHRF1BP1, EEA1, HOOK3, AP3S1, CHD9, UTP18, KCNQ5, C20orf112, MAST4, ETS1, RNF114, TOP1, CNTNAP2, ENAH, ZEB2, ACBD5, MAMDC2, JPH1, PURB, VPS13A, SPAST, TBC1D15, PTPN14, PIM1, LMX1A, KCNN3, PFN2, H2AFZ, C1orf151/MINOS1, ROD1/PTBP3*

***miR-708-5p*:**

*NRAS, CREB1, HNRNPC, PSMF1, RPGRIP1L, ATXN1, ALG9, RFT1, PFKM, QKI, GRIP2, AAK1, FOXN3, SLC44A5, STK4, LIF, MDGA1, AS3MT, FAM135B, BSN, ETF1, CDC42SE1, SHANK3, C20orf112, INTS7, GLG1, RORA, SSH2, ITPKB, SLC39A14, KPNA4, RNF165, HS2ST1, KCNN3, GOLT1A, C1orf144/SZRD1*

***miR-1908-5p*:**

*KLC2, NCDN, MDGA1, SLC12A5, HPD, NRGN, MINK1, PCDHA1, GDF11, OTX1, EEF1A2, SLC17A7*
